# State of the art and clinical recommendations in periapical implant lesions. 9th Mozo-Grau Ticare Conference in Quintanilla, Spain

**DOI:** 10.4317/jced.53600

**Published:** 2017-03-01

**Authors:** Miguel Peñarrocha-Diago, María Peñarrocha-Diago, Juan-Antonio Blaya-Tárraga

**Affiliations:** 1 MD, MDM, PhD, Professor and Chairman of Oral Surgery, Stomatology Department, Faculty of Medicine and Dentistry, University of Valencia, Spain; 2 MD, DMD, PhD, Assistant Professor of Oral Surgery, Stomatology Department, Faculty of Medicine and Dentistry, University of Valencia, Spain; 3 DDS, MSc. Master in Oral Surgery and Implantology. Faculty of Medicine and Dentistry, University of Valencia, Spain

## Abstract

This manuscript summarizes the statements and clinical recommendations in periapical implant lesions, as per the state of the art and expert opinion agreement among the participants in the 9th Mozo-Grau Conference 2016 held in Quintanilla (Valladolid, Spain). The current status of the concept, frequency, etiology, diagnosis, clinical classification, surgical procedure and prognosis are described. If following implant placement localized pain develops in the periapical area, with or without radiographic changes, the diagnosis of periapical implant lesion should be suspected. It is important to monitor the condition in order to identify any change in its evolution. Radiological changes in the periapical radiographs are not always manifest in the early stages, and in this regard small-volume cone beam computed tomography can help us visualize such peri-implant changes. The early diagnosis of periapical implant lesions during the osseointegration phase and the provision of early treatment result in increased implant survival rates, thereby avoiding the need for implant extraction.

** Key words:**Apical peri-implantitis, retrograde peri-implantitis, inflammatory peri-implantitis lesion.

## Introduction

Periapical implant lesions, also referred to as apical peri-implantitis or retrograde peri-implantitis, were first described by McA-llister in 1992 (1) as injuries in the apical portion of implants, causing osseointegration failure. Sussman & Moss (2) defined the disorder as an infectious-inflammatory process of the tissues surrounding the implant apex, while Quirynen *et al.* (3) described it as a clinically symptomatic periapical lesion that develops shortly after implant insertion while the coronal portion of the implant achieves a normal bone-to-implant interface.

This report summarizes the statements and clinical recommendations in periapical implant lesions, as per the state of the art and expert opinion agreement among the participants in the 9th Mozo-Grau Conference 2016 held in Quintanilla (Valladolid, Spain).

The etiology of the lesion is not yet clear; however, several factors have been proposed that could be related to the onset of the disorder. According to some authors, the most likely cause is endodontic disease of the tooth replaced by the implant or of the adjacent tooth (4-8). Other described factors are contamination of the implant surface (9,10), bone overheating during milling or preparation exceeding that required for the implant (9,11,12), pre-existing bone disease, and the presence of root fragments or foreign bodies (5,9,12).

The present report assesses the literature to describe the concept, frequency, etiology, diagnosis, clinical classification, surgical procedure and prognosis of the disorder. Early diagnosis and treatment result in increased implant survival rates, thereby avoiding the need for implant extraction.

Periapical implant lesions: A systematic review

-Focus question (PEO)

“In patients with periapical implant lesions during osseointegration, what symptoms, signs, and changes in complementary examination develop recommending appropriate management in this stage?”

-Consensus statements: State of the art and clinical recommendations.

-Concept

Different nomenclatures have been proposed: apical / periapical implant lesion, retrograde peri-implantitis or early peri-implantitis. A difficulty in the systematic review was to differentiate between articles describing coronal or apical peri-implantitis.

Therefore, the consensus group proposes the definition of this condition as a periapical implant lesion of inflammatory and infectious nature, developing in the axial axis of the implant during osseointegration, with the maintenance of normal coronal bone in early stages.

-Frequency

The frequency of periapical implant lesions shows considerable discrepancies between studies, ranging from 0.26% to 2.7%. In implants with adjacent teeth subjected to endodontic treatment, the incidence can reach 7.8%. The frequency of this condition is low - a fact that may be attributed to lack of knowledge and insufficient study of disorder. Studies involving larger patient samples are needed to provide more data on the frequency of periapical implant lesions.

-Etiology

The cause of early loss of well placed implants is not clear. Different etiological factors for periapical implant lesions have been suggested, though the evidence is very limited. The factors can be grouped according to the source of contamination as follows:

a) Contamination of the surgical bed: implant surface contamination (saliva, epithelial cells or lubricant oil from rotary instruments), the surgical bed itself, the presence of remnants of milling or overheating of bone during drilling.

b) Pre-existing disease: immediate post-extraction placement, endodontic pathology associated to the extracted tooth or adjacent teeth (there is no evidence referred to the distance between teeth and implants, though this factor is important for the development of such infections), pre-existing bone disease, and the presence of root fragments or foreign bodies.

All factors derived from surgery and the patient are important for controlling tissue healing, and although oral surgery constitutes non-aseptic surgery, it is important to follow aseptic protocols at all times.

-Diagnosis

The diagnosis of periapical implant lesions involves clinical and radiographic assessments. The symptoms (pain and puffiness) and signs (swelling, fistula and drainage) may appear with variable intensity depending on the stage of the lesion. Radiographically, a radiolucency around the implant apex may be observed. Peri-implant radiolucencies due to over-drilling may be casual findings during routine radiographic assessments. The implementation of new imaging technologies such as small-volume cone beam computed tomography (CBCT) is of help in establishing an early diagnosis, showing a clear clinical image of periapical implant bone loss. As a complement to periapical radiographs, small-volume CBCT, in addition to exploratory surgery, can be used in cases of difficult diagnosis. In order to establish a correct diagnosis of this type of lesion, radiographic protocol-based monitoring is recommended from the time of implant placement.

Regarding the time at which this condition is detected, the radiological findings usually appear between 7 and 16 days after surgery, and until three months after implant placement.

The surgeon is that which has the perception of not having fenestration during surgery, prior consideration of the integrity of all bony walls is advised. Discarded conditions are: iatrogenic (for milling), adjacent or residual lesions, surgeries guided bone regeneration (pain may be due to contamination of biomaterial).

-Clinical classification

Because of the difficulty in establishing an objective diagnosis, it is considered that the current classifications do not cover all possible factors. Could be established individual classifications to the injury taking into account the origin of infection.

-Treatment

Treatment is based on the clinical diagnosis and findings of the radiological explorations, with individualized assessment of the periapical implant lesion and systemic conditions of the patient. If the implant has a radiolucent area (not present after surgery due to over-drilling and manifesting over time), without pain, monitoring of the lesion is recommended, without medical treatment. If the radiolucency has increased in size or if the patient develops pain, medical and surgical treatment is indicated.

-Surgical procedure 

Surgical treatment comprises infiltrative anesthesia, incision, raising of a full-thickness flap, osteotomy, periapical curettage of granulation tissue, profuse irrigation with sterile saline solution and tensionless flap closure with monofilament suture.

Although described, apical implant sectioning is not considered necessary: with the new curettes and ultrasonic tips, removal of granulation tissue and curettage of the residual bone cavity is ensured.

The use of bone substitutes for bone regeneration of the defect is not advised. It might be interesting to place a collagen membrane, depending on the bone defect, in order to avoid soft tissue infiltration in the apex of the implant and improve new bone formation in the cavity.

Prophylactic antibiotic treatment is recommended, continuing in the postoperative period for at least one week, and combining a broad spectrum antibiotic such as amoxicillin with another drug effective against anaerobes, such as metronidazole.

-Prognosis

The prognosis referred to these lesions is favorable - the literature reporting survival rates of 73.2% to 97.4% of the implants trea-ted with a maximum follow-up of 10.5 years. Success depends on early diagnosis and adequate remaining bone fixation. The study of periapical radiographs and CBCT will improve our ability to diagnose inflammatory disease of the implant.

-General clinical recommendations:

• Preoperatively, emphasis is placed on the need to assess the periodontal and endodontic condition of the adjacent teeth, the teeth to be removed, and the receptor bone.

• As a preventive measure, profuse irrigation with sterile saline solution during the drilling sequence and in the final surgical bed is advised, though supporting scientific evidence is lacking.

• If localized pain develops in the periapical area after implant placement, with or without associated radiographic changes, the diagnosis of periapical implant lesion should be suspected.

• It is important to monitor the situation in order to identify any change in evolution. In the initial phases radiological changes are not always seen on the periapical radiographs. In this regard, small-volume CBCT can help us to visualize changes in the peri-implant area.

•The early diagnosis of periapical implant lesions during the osseointegration phase and the early treatment result in increased implant survival rates, thereby avoiding the need for implant extraction.

-General recommendations for future research:

• Design prospective studies.

• Adoption of a homogeneous protocol for the collection of clinical variables.

• Application of a data collection protocol during the osseointegration phase for future studies.
